# Case Series of Acute Appendicitis Associated With Waterborne Pathogen Exposure in College Students

**DOI:** 10.7759/cureus.77086

**Published:** 2025-01-07

**Authors:** Mohamed Y Ali, Yassin A Mohamedali, Hassan K Mohammed, Ismaeel M Elzaki, Ali A Ali, Mohamed J Belo

**Affiliations:** 1 Surgery, Damazin Teaching Hospital, Ad-Damazin, SDN; 2 Medicine, Tadawi Medical Center, Abu Dhabi, ARE; 3 Medicine, University of Khartoum, Khartoum, SDN

**Keywords:** appendicitis, e. coli, gastrointestinal diseases, polluted water, pseudomonas aeruginosa, waterborne pathogens, yersinia enterocolitica

## Abstract

This case series investigates the incidence of acute appendicitis among 24 college students in Ad-Damazin, Blue Nile region, Sudan, linked to the consumption of polluted water. This study was conducted from December 21, 2023, to January 4, 2024, with patients presenting to the emergency department exhibiting symptoms consistent with appendicitis. Diagnostic confirmation was achieved through clinical evaluation, radiological imaging, and histopathological examination. Pathogens isolated included *Pseudomonas aeruginosa* (54.2%), *Escherichia coli* (20.8%), and *Yersinia enterocolitica* (8.3%), correlating with pathogens detected in contaminated water samples. Patients with confirmed appendicitis experienced significant morbidity, including surgical site infections in two cases, which extended their hospitalization. Following water source decontamination, no new cases of appendicitis were reported, supporting a direct link between polluted water and appendicitis incidence. This study highlights the role of environmental factors in appendicitis etiology and underscores the necessity for improved water quality management in high-risk areas. Public health interventions targeting water sanitation could significantly reduce appendicitis cases and related complications, emphasizing the need for further research on environmental influences on gastrointestinal health.

## Introduction

Acute appendicitis is the most common non-trauma surgical emergency, with a peak incidence in the second and third decades of life and an estimated lifetime risk of 7-8% in the general population [1,2]. Although acute appendicitis has been studied extensively, its pathogenesis is still a matter of debate.

Acute appendicitis is believed to result from obstruction of the appendiceal lumen, which may be due to lymphoid hyperplasia, fecaliths, or foreign bodies. This obstruction can lead to increased intraluminal pressure, bacterial overgrowth, and inflammation [3]. It could also result from non-obstructive causes, such as bacterial and viral infections [4].

While dietary habits, lifestyle factors, and genetic predispositions are well-documented contributors to appendicitis risk, recent studies have suggested that environmental exposures, particularly to polluted drinking water, may also influence the incidence of appendicitis [5]. Polluted water is defined as water contaminated with chemicals or microorganisms that can cause adverse biological effects in resident communities [6]. Consumption of polluted water introduces a range of pathogenic bacteria into the gastrointestinal system, which may trigger inflammation or infection of the appendix. Notably, waterborne pathogens such as *Pseudomonas aeruginosa*, *Escherichia coli (E. coli)*, Yersinia, *Salmonella typhi*, and Clostridium species have been identified as potential contributors to appendiceal infections due to their ability to colonize and inflame the gastrointestinal tract, with *E. coli* and *Pseudomonas aeruginosa* being the most common microorganisms to cause appendicitis [7-9].

*Escherichia coli* is a commensal Gram-negative, rod-shaped bacterium commonly found in the gut of humans and warm-blooded animals. Most *E. coli* species are considered harmless to humans. However, certain pathogenic strains can produce toxins that damage the intestinal lining, promoting local inflammation and increasing the risk of appendicitis.

*Pseudomonas aeruginosa* is a Gram-negative, aerobic, non-spore-forming rod that can cause gastrointestinal infections leading to localized inflammation and tissue damage. Studies suggest that Pseudomonas biofilm formation in the appendix could contribute to inflammation, potentially increasing the risk of acute appendicitis [10,11]. *Yersinia enterocolitica* is also associated with gastrointestinal infections. It is known to mimic appendicitis symptoms due to its tendency to infect the ileum and lymphoid tissues near the appendix, and in some cases cause appendicitis [7].

*Salmonella typhi* is a Gram-negative, obligate anaerobic bacterium known to cause typhoid fever, as well as systemic and gastrointestinal disorders. It has been found that this bacterium has the ability to invade the intestinal mucosa, triggering inflammation and swelling in the appendix, making it one of the causes of acute appendicitis [9].

Clostridium species, including *Clostridium difficile*, are commonly present in the environment and can cause severe gastrointestinal conditions. Some strains of Clostridium release toxins that lead to inflammation and disruption of the gut microbiome. Evidence indicates that exposure to these bacteria may contribute to appendiceal infection [12].

Given the potential environmental risk factors, this study aimed to investigate the incidence of a series of cases among 24 college students who consumed polluted water and presented to Damazin Teaching Hospital Emergency Department with acute abdominal pain in the period from December 21, 2023, to January 4, 2024, and to examine the association between consumption of polluted water containing *Pseudomonas aeruginosa*, *E. coli*, Yersinia, and Clostridium species and the development of acute appendicitis. Understanding such associations could contribute to preventative public health measures, potentially reducing the incidence and complications of acute appendicitis in high-risk populations. This study highlights the environmental factors role in appendicitis etiology and underscores the necessity for improved water quality management in high-risk areas.

## Materials and methods

Study design

This case series study examines the acute appendicitis cases linked to waterborne pathogens exposure among college students in Ad-Damazin, Blue Nile region, Sudan. Cases included in this study were identified as patients who presented to the Emergency Department at Damazin Teaching Hospital between December 21, 2023, and January 4, 2024. Diagnosis was done through clinical, radiological, and laboratory investigations, and confirmation through intraoperative observation, histopathology, and microbiological analysis.

Patients with confirmed acute appendicitis were grouped based on bacterial culture results and histopathological examination findings, with subgroups created for bacterial-positive cases and bacterial-negative cases. Only cases with documented exposure to potentially polluted water sources were included.

Inclusion and exclusion criteria

Inclusion criteria were college students diagnosed with acute appendicitis and with potential exposure to untreated or inadequately treated water sources. Students were excluded if they did not have reliable exposure information or presentation not related to the consumption of polluted water.

Data collection

Data were collected through medical record reviews and structured interviews with affected students. Information on demographic factors, water exposure history, and clinical presentation were obtained. To establish a baseline, additional details about the students' living conditions and sanitation practices were recorded.

Statistical analysis

Descriptive statistics were used to summarize the demographic and clinical characteristics of the study population. Proportions were calculated for categorical variables, including the percentage of patients presenting with specific symptoms, signs, and pathogens identified in both appendiceal and water samples. Hospital stay duration and occurrence of SSIs were analyzed as indicators of severity.

Intraoperative macroscopic findings

The most common finding in appendiceal characteristics was hyperemic and edematous appendices, which were observed in 22 patients (91.7%). The appendices appeared swollen and intensely reddened, consistent with acute inflammation. Necrosis at the base of the appendix was noted in one patient (4.2%), indicating a more severe and advanced stage of appendicitis. Regarding peritoneal and pelvic findings, minimal pelvic fluid collection was detected in 15 patients (62.5%), often described as clear or slightly cloudy fluid. This reflects localized inflammation and early peritoneal involvement. Pus collection was identified in one patient (4.2%), highlighting localized infection around the appendix. Erythematous ileum was observed in one patient (4.2%), suggesting localized irritation or inflammation of adjacent bowel structures. In two patients (8.3%) there was no intraoperative evidence of appendicitis; the appendices appeared normal, with no signs of hyperemia, edema, or inflammation. These findings were later confirmed by histopathology as non-inflamed appendices.

## Results

This case series included 24 patients (college students) who presented with acute abdominal pain to Damazin Teaching Hospital Emergency Department with acute abdominal pain in the period from December 21, 2023, to January 4, 2024, after consuming polluted water. All individuals exhibited symptoms and signs consistent with acute appendicitis, leading to surgical intervention. Six patients (25%) presented within 24 hours of symptom onset, 15 patients (62.5%) presented within 48 hours, and three patients (12.5%) presented after 48 hours.

Common symptoms

The patients in this case series exhibited a range of symptoms commonly associated with acute appendicitis. Central abdominal pain was noted in 10 patients (41.7%), while right lower quadrant (RLQ) pain affected 14 patients (58.3%). Nausea was prevalent, occurring in 20 patients (83.3%), and vomiting was reported by 10 patients (41.7%). Additionally, five patients (20.8%) experienced diarrhea, and eight patients (33.3%) had anorexia. Together, these symptoms highlighted the acute presentation and were consistent with the diagnosis of appendicitis.

Physical examination findings

On physical examination, the majority of patients displayed notable signs consistent with acute appendicitis. Fever was recorded in 22 patients (91.7%), and tachycardia was noted in 20 patients (83.3%). Right iliac fossa tenderness, a key indicator of appendicitis, was present in 23 patients (95.8%). Positive rebound tenderness was observed in 18 patients (75%), while six patients (25%) showed a positive psoas sign. These examination findings collectively reinforced the diagnosis of acute appendicitis.

Alvarado score

The Alvarado score is a clinical tool used to diagnose acute appendicitis by assessing symptoms, signs, and laboratory findings. It helps determine the likelihood of appendicitis by stratifying patients into low-, moderate-, or high-risk categories [13]. The Alvarado scores for the patients in this case series ranged from 7 to 10, reflecting a high likelihood of acute appendicitis in the cohort. Specifically, five patients (20.8%) had a score of 7, while 10 patients (41.7%) scored 8. A score of 9 was observed in five patients (20.8%), and four patients (16.7%) had a maximum score of 10. These high Alvarado scores supported and confirmed the clinical assessment of appendicitis.

Surgical findings

Intraoperative Diagnosis

Macroscopic features intraoperatively suggesting acute appendicitis were noticed in 22 patients (91.7%), characterized by hyperemic and edematous appendices, with minimal pelvic fluid collection noted in many cases (Table 1). Necrosis in the base of the appendix was noted in one of them. Two patients did not show features of appendicitis (8.3%). We grouped the cases based on pathogen identification, histopathology findings, and complications.

**Table 1:** Presentation of the included cases. RLQ: right lower quadrant; RIF: right iliac fossa; +ve: positive; -ve: negative; PMH: past medical history; *E. coli: Escherichia coli*; Pt: patient

Patients number	Age (years)	Gender	PMH	Presentation	Examination findings	Alvarado score	Intraoperative findings	Symptoms onset before presentation	Duration of hospitalization	Laboratory findings
1	20	Female	Clear	Central abdominal pain, nausea	Fever, tachycardia, RIF tenderness, +ve rebound tenderness	8	Minimal pelvic fluid collection, hyperemic appendix	2 days	5 days	Acute appendicitis, swab +ve for *Pseudomonas aeruginosa*, Pt developed SSI +ve for the same organism
2	22	Female	Clear	RLQ pain, nausea, diarrhea, vomiting	Fever, RIF tenderness, +ve rebound tenderness, +ve psoas sign	7	Necrosis at the base of appendix, erythematous ilium	3 days	2 days	Acute appendicitis, swab +ve for *Yersinia enterocolitica*
3	22	Female	Clear	RLQ pain, nausea, anorexia, migration of pain	Fever, RIF tenderness, +ve rebound tenderness	8	Minimal pelvic fluid collection, hyperemic edematous appendix	2 days	5 days	Acute appendicitis, swab +ve for *Pseudomonas aeruginosa*, Pt developed SSI +ve for the same organism
4	20	Female	PMH of tonsillectomy	RLQ pain, nausea, migration of pain	Fever, RIF tenderness, +ve rebound tenderness	8	Minimal pelvic fluid collection, hyperemic edematous appendix	2 days	4 days	Acute appendicitis, swab +ve for *Pseudomonas aeruginosa*
5	20	Female	Clear	Central abdominal pain, nausea, anorexia, diarrhea	RIF tenderness, +ve rebound tenderness	7	Minimal pelvic collection	2 days	2 days	Normal appendix
6	19	Female	Clear	Central abdominal pain, nausea, vomiting	Fever, RIF tenderness, +ve rebound, tenderness, +ve psoas sign	8	Hyperemic edematous appendix, with minimal pus collection	4 days	2 days	Acute appendicitis, awab +ve for *E. coli*
7	20	Female	Clear	Central abdominal pain, nausea, vomiting	Fever, RIF tenderness, +ve rebound tenderness	8	Hyperemic edematous appendix	2 days	2 days	Acute appendicitis, swab +ve for *E. coli*
8	21	Female	Clear	RLQ pain, nausea, migration of pain, anorexia	Fever, RIF tenderness, +ve rebound tenderness	10	Hyperemic edematous appendix	2 days	3 days	Acute appendicitis, swab +ve for *Pseudomonas aeruginosa*
9	21	Female	FH of similar condition (brother)	RLQ pain, nausea, migration of pain	RIF tenderness, +ve rebound tenderness	8	No intraoperative findings	2 hours	2 days	Normal appendix
10	26	Female	Clear	RLQ pain, nausea	Fever, RIF tenderness, +ve rebound tenderness	7	Minimal pelvic fluid collection, hyperemic edematous appendix	2 days	3 days	Acute appendicitis, swab +ve for *Pseudomonas aeruginosa*
11	22	Female	Clear	RLQ pain, nausea, migration of pain	Fever, RIF tenderness, +ve rebound tenderness	10	Minimal pelvic fluid collection, hyperemic edematous appendix	2 days	2 days	Acute appendicitis, swab +ve for *Pseudomonas aeruginosa*
12	18	Female	Clear	Central abdominal pain, nausea, vomiting	Fever, RIF tenderness, +ve rebound tenderness	7	Minimal pelvic fluid collection, hyperemic edematous appendix	2 hours	2 days	Acute appendicitis, -ve swab
13	19	Female	Clear	RLQ pain, nausea, migration of pain	Fever, RIF tenderness, +ve rebound tenderness	8	Minimal pelvic fluid collection, hyperemic edematous appendix	2 days	2 days	Acute appendicitis, swab +ve for *E. coli*
14	24	Male	Clear	RLQ pain, nausea, vomiting	Fever, RIF tenderness, +ve rebound tenderness	8	Hyperemic edematous appendix	3 hours	2 days	Acute appendicitis, swab +ve for *Pseudomonas aeruginosa*
15	20	Female	Clear	Central abdominal pain, nausea, anorexia	Fever, RIF tenderness, +ve rebound tenderness	9	Hyperemic edematous appendix	5 hours	2 days	Acute appendicitis, swab +ve for *Pseudomonas aeruginosa*
16	19	Female	Clear	RLQ pain, nausea, diarrhea, migration of pain	Fever, RIF tenderness, +ve rebound tenderness	8	Hyperemic edematous appendix	2 days	2 days	Acute appendicitis, -ve swab
17	22	Male	Clear	RLQ pain, nausea, vomiting	RIF tenderness, +ve rebound tenderness	7	Hyperemic edematous appendix	2 days	3 days	Acute appendicitis, swab +ve for *Pseudomonas aeruginosa*
18	20	Female	Clear	RLQ pain, nausea, vomiting	Fever, RIF tenderness, +ve rebound tenderness	8	Hyperemic edematous appendix	3 days	2 days	Acute appendicitis, swab +ve for *Yersinia enterocolitica*
19	20	Female	Clear	RLQ pain, nausea	Fever, RIF tenderness, +ve rebound tenderness	8	Hyperemic edematous appendix	2 days	2 days	Acute appendicitis, swab +ve for *Pseudomonas aeruginosa*
20	19	Female	Clear	Central abdominal pain, nausea, anorexia	Fever, RIF tenderness, +ve rebound tenderness	9	Hyperemic edematous appendix	2 hours	2 days	Acute appendicitis, swab +ve for *Pseudomonas aeruginosa*
21	20	Female	Clear	RLQ pain, nausea, vomiting	Fever, RIF tenderness, +ve rebound tenderness	8	Hyperemic edematous appendix	2 days	2 days	Acute appendicitis, swab +ve for *E. coli*
22	20	Female	Clear	RLQ pain, nausea, vomiting	Fever, RIF tenderness, +ve rebound tenderness	8	Hyperemic edematous appendix	7 hours	2 days	Acute appendicitis, swab +ve for *E. coli*
23	21	Female	Clear	RLQ pain, nausea	Fever, RIF tenderness, +ve rebound tenderness	8	Hyperemic edematous appendix	2 days	3 days	Acute appendicitis, swab +ve for *Pseudomonas aeruginosa*
24	20	Female	Clear	RLQ pain, nausea, diarrhea	Fever, RIF tenderness, +ve rebound tenderness	8	Hyperemic edematous appendix	2 days	2 days	Acute appendicitis, swab +ve for *Pseudomonas aeruginosa*

Group 1: This group included cases with confirmed acute appendicitis and positive bacterial cultures (n=20). Among the 24 patients, 20 had histopathology-confirmed acute appendicitis with bacterial pathogens isolated from surgical swabs. In this group, three pathogens were identified which include the following: (1) *Pseudomonas aeruginosa* (13 patients; 54.2%), patients in this group commonly presented with right lower quadrant (RLQ) pain, fever, and elevated Alvarado scores between 7 and 10 (average of 8.3). Most required hospital stays of two to three days. Two of these patients developed surgical site infections (SSIs), with *Pseudomonas aeruginosa* isolated from both intraoperative and postoperative swabs. Which increased their hospitalization period, the mean duration of hospitalization for patients who developed SSIs was five days, compared to an average of 2.3 days for non-SSI cases. (2) *Escherichia coli* (five patients; 20.8%), patients with *E. coli* infections generally presented similarly, with RLQ pain, fever, and positive rebound tenderness. The average hospital stay in this subgroup was 2.3 days. (3) *Yersinia enterocolitica* (two patients; 8.3%), these patients exhibited appendiceal necrosis form intraoperatively. The hospital stays averaged two days.

Group 2: This group included cases with confirmed acute appendicitis and negative bacterial cultures (n=2). Two patients presented with RLQ pain and central abdominal pain, fever, a high Alvarado score (average of 8.5), and were found to have acute appendicitis based on histopathology. However, no pathogens were isolated in their intraoperative swab cultures. They had intraoperative findings of hyperemic and edematous appendices. Patients in this group had an average stay of two days.

Group 3: This group included cases with normal appendices on histopathology (n=2). Two patients had negative histopathology findings, with normal appendices and no bacterial pathogens identified on culture. These patients also presented with RLQ pain and fever, and Alvarado scores of 8 each. However, intraoperative examination revealed no features suggesting acute appendicitis, histopathology confirmed the absence of inflammation, and both swabs came back negative. Both patients had shorter hospital stays, one day for both of them.

Water Sample Analysis and Postintervention Findings

To investigate the potential link between polluted water consumption and the development of acute appendicitis, we conducted an analysis of 10 water samples collected from the sources consumed by the participants. The samples were analyzed using both aerobic and anaerobic methods, including the use of blood agar, MacConkey agar, and cystine-lactose-electrolyte-deficient (CLED) agar. Additionally, Gram staining, peptone broth media, as well as indole and oxidase tests were performed to identify the presence of pathogens.

The results of the water analysis revealed the following pathogenic organisms: *Pseudomonas aeruginosa *was detected in six out of 10 samples, indicating a significant presence of this pathogen in the water supply, *Escherichia coli *was isolated from four samples, suggesting contamination with fecal material, *Yersinia enterocolitica *was identified in two samples and, *Clostridium difficile *was found in two samples.

The identification of these pathogens, particularly *Pseudomonas aeruginosa* and *Escherichia coli*, aligns with the clinical presentations and histological and microbiological analysis of the students, supporting the hypothesis that exposure to polluted water and associated waterborne pathogens may be linked to the development of acute appendicitis. Following these findings, the suspected water source underwent thorough decontamination. Notably, no additional cases of appendicitis associated with the contaminated water were reported, further supporting the hypothesis.

## Discussion

This study presents a unique case series linking acute appendicitis in college students to the consumption of polluted water with specific pathogens such as *Pseudomonas aeruginosa*, *Escherichia coli (E. coli)*, and *Yersinia enterocolitica* isolated from both water samples and patients’ specimens. The results highlight a possible association between waterborne pathogen exposure and appendicitis risk, emphasizing the need to consider environmental factors, particularly water quality, as part of appendicitis etiology.

Environmental factors and appendicitis incidence

Traditionally, appendicitis is thought to arise from luminal obstruction, often caused by fecaliths, lymphoid hyperplasia, or foreign bodies, leading to inflammation and bacterial overgrowth within the appendix [3]. However, recent studies indicate that microbial infections, particularly from pathogenic bacteria, can play a significant role in appendiceal inflammation without an obstructive cause [4].

The emerging role of environmental exposures, specifically polluted water, represents an area of interest and growing concern. Polluted water, containing pathogenic bacteria, can disrupt the gastrointestinal microbiome, promote inflammation, and potentially lead to appendicitis. Our findings align with previous research suggesting that waterborne pathogens might contribute to the onset of appendicitis, either by direct infection or by creating conditions favorable for bacterial overgrowth and inflammation [5].

Role of identified pathogens

The predominance of *Pseudomonas aeruginosa* and *E. coli* among isolated pathogens highlights these bacteria's roles in appendicitis development. *Pseudomonas aeruginosa*, identified in 54.2% of patients with confirmed appendicitis, is known for its biofilm formation, which can enhance bacterial persistence and induce chronic inflammation within the gastrointestinal tract. Recent studies suggest that biofilm-forming bacteria, such as *Pseudomonas aeruginosa*, may exacerbate inflammatory responses, potentially leading to appendicitis, as noted in the present study with patients who presented with high Alvarado scores and also patients who had postoperative complications - surgical site infections (SSI) - extending their hospital stays significantly compared to non-SSI cases (averaging five days versus 2.3 days for non-SSI patients) [7,10].

*E. coli*, which was isolated in 20.8% of confirmed cases, is known for producing toxins that disrupt the gut lining and lead to local inflammation and it’s the most common bacteria associated with cases of acute appendicitis [8,14]. The detection of Yersinia enterocolitica in a smaller subset of patients (8.3%) is consistent with findings that associate this bacterium with inflammation of the ileocecal region, which can mimic or trigger appendicitis-like symptoms (pseudoappendicitis) and, in some cases, maybe a cause of appendicitis [7,15].

Clinical and surgical findings

The presentation patterns observed in this study, particularly fever, right iliac fossa tenderness, rebound tenderness, and elevated Alvarado scores, are consistent with the typical diagnostic profile for appendicitis. The variations in symptom onset among patients - ranging from 24 hours to over 48 hours - suggest variability in individual immune responses to infection, possibly influenced by the specific pathogen involved. Surgical findings across cases - hyperemic, edematous appendices, and occasional necrosis - further support the link between waterborne pathogen exposure and acute appendicitis.

Implications for public health and preventative measures

This case series highlights critical public health implications, particularly in regions where water quality is compromised. The findings reinforce the need for effective water sanitation and hygiene measures to reduce bacterial contamination, as interventions to clean the water source post-study led to a noticeable cessation of new appendicitis cases associated with the consumption of polluted water. Studies in regions with similar environmental risks have reported that access to clean water can significantly reduce not only gastrointestinal infections but also associated complications, such as appendicitis, further underscoring the potential benefits of water quality interventions [16]. These findings suggest that public health initiatives to prevent water contamination may be an effective preventative measure against certain cases of acute appendicitis in high-risk areas.

Comparison with similar studies

Similar studies have been conducted in other regions, identifying correlations between waterborne pathogen exposure and incidence of GI disorders including appendicitis. A retrospective study in rural regions of Southeast Asia, for example, found that communities with high levels of waterborne *E. coli *and *Pseudomonas aeruginosa *contamination had a higher incidence of appendicitis and other abdominal infections, supporting our findings [17]. Additionally, research conducted in North Africa reported that polluted water with similar pathogenic profiles was associated with increased GI disorder cases, particularly in areas lacking infrastructure for safe water provision [18]. These studies reinforce the need to consider local water quality as part of appendicitis etiology and encourage further research in other developing regions with high water contamination.

Study strengths, limitations, and recommendations

The strengths of this study, along with its public health relevance, lie in its unique context and focus. It examines the association between waterborne pathogens and acute appendicitis, providing valuable insights into a potentially underexplored aspect of appendicitis etiology. The successful isolation and identification of specific pathogens (*Pseudomonas aeruginosa*, *Escherichia coli*, and *Yersinia enterocolitica*) from both water and patient specimens strengthens the argument that waterborne pathogens may contribute to the development of appendicitis. Additionally, the clinical and surgical data collected, including Alvarado scores, physical examination findings, and detailed intraoperative observations, offer a clear and comprehensive picture of the clinical presentation and progression of acute appendicitis in this cohort.

The study's limitations include a relatively small sample size (24 patients). As an observational case series, it cannot establish causality between waterborne pathogen exposure and appendicitis. Although the correlation is strong, further research is needed. Additionally, there is a lack of long-term follow-up data on patient recovery after discharge, recurrence of appendicitis, or the long-term impact of improved water sanitation on health outcomes.

Further studies with larger populations and prospective designs are warranted to explore this association more comprehensively. Additionally, research into potential bacterial mechanisms, such as biofilm formation and toxin production, could provide insights into how waterborne pathogens directly contribute to appendicitis. Future research might also investigate the roles of other potential pathogens, such as *Clostridium difficile* and *Salmonella typhi*, which, while not prominently identified in our cases, have been associated with inflammation and infections in similar environmental studies.

## Conclusions

This case series demonstrates a probable link between polluted water consumption and acute appendicitis in college students in Al-Damazin, Blue Nile region. Pathogens isolated from both patients and water samples, including *Pseudomonas aeruginosa, Yersinia enterocolitica, *and *Escherichia coli*, underscore the role of waterborne pathogens in appendicitis risk. The absence of new similar cases following water decontamination suggests that clean water interventions are critical for preventing similar health risks. Further studies in resource-limited areas with comparable environmental exposures are essential to reinforce these findings and inform preventive public health strategies.
